# Full-body kinematics and head stabilisation strategies during walking in patients with chronic unilateral and bilateral vestibulopathy

**DOI:** 10.1038/s41598-024-62335-1

**Published:** 2024-05-23

**Authors:** Gautier Grouvel, Anissa Boutabla, Julie Corre, Rebecca Revol, Marys Franco Carvalho, Samuel Cavuscens, Maurizio Ranieri, Jean-François Cugnot, Christopher McCrum, Raymond van de Berg, Nils Guinand, Angélica Pérez Fornos, Stéphane Armand

**Affiliations:** 1 Division of Otorhinolaryngology Head and Neck Surgery, Geneva University Hospitals and University of Geneva, Geneva, Switzerland; 2 Kinesiology Laboratory, Geneva University Hospitals and University of Geneva, Geneva, Switzerland; 3 Division of Clinical Neurosciences, Geneva University Hospitals, Geneva, Switzerland; 4 Department of Nutrition and Movement Sciences, NUTRIM School of Nutrition and Translational Research in Metabolism, Maastricht University Medical Center+, Maastricht, The Netherlands; 5 Division of Balance Disorders, Department of Otorhinolaryngology and Head and Neck Surgery, Maastricht University Medical Center+, Maastricht, The Netherlands

**Keywords:** Diagnostic markers, Movement disorders, Neurological disorders

## Abstract

Chronic imbalance is a frequent and limiting symptom of patients with chronic unilateral and bilateral vestibulopathy. A full-body kinematic analysis of the movement of patients with vestibulopathy would provide a better understanding of the impact of the pathology on dynamic tasks such as walking. Therefore, this study aimed to investigate the global body movement during walking, its variability (assessed with the GaitSD), and the strategies to stabilise the head (assessed with the head Anchoring Index). The full-body motion capture data of 10 patients with bilateral vestibulopathy (BV), 10 patients with unilateral vestibulopathy (UV), and 10 healthy subjects (HS) walking at several speeds (slow, comfortable, and fast) were analysed in this prospective cohort study. We observed only a few significant differences between groups in parts of the gait cycle (shoulder abduction–adduction, pelvis rotation, and hip flexion–extension) during the analysis of kinematic curves. Only BV patients had significantly higher gait variability (GaitSD) for all three walking speeds. Head stabilisation strategies depended on the plan of motion and walking speed condition, but BV and UV patients tended to stabilise their head in relation to the trunk and HS tended to stabilise their head in space. These results suggest that GaitSD could be a relevant biomarker of chronic instability in BV and that the head Anchoring Index tends to confirm clinical observations of abnormal head-trunk dynamics in patients with vestibulopathy while walking.

## Introduction

Vestibular disorders have a worldwide prevalence of 5 to 10% in the population over the age of 65^1^ and have a significant impact on quality of life. Correct and rapid diagnosis remains a challenge^2–4^. Symptoms in vestibular disorders result from pathological fluctuations of the peripheral or central vestibular system or from the chronic loss of vestibular function. Chronic loss of vestibular function, also called vestibulopathy, can be progressive or abrupt, depending on the aetiology, such as ototoxic, infectious, traumatic, or congenital^2^. The dysfunction may affect one ear (unilateral vestibulopathy) or both ears (bilateral verstibulopathy^5^).

Bilateral vestibulopathy (BV) is a heterogeneous chronic condition characterised by a bilaterally reduced or absent function of the vestibular organs and/or the vestibular nerves^6^. Although BV patients mainly complain of imbalance and oscillopsia, a majority although reports visual vertigo, having problems with dual tasking or spatial orientation^2,7^. In contrast, unilateral vestibulopathy (UV) is a heterogeneous condition that partially or totally affects the function of the vestibular organs and/or nerves unilaterally^8^. This loss can occur suddenly or gradually depending on the aetiology^8^. The principal symptoms that affect UV patients are dizziness, imbalance, and oscillopsia^8^.

The peripheral vestibular system, composed of a pair of 3 semicircular canals and a pair of 2 otolith organs, acts as a motion sensor, detecting movements of the head^9^. It generates very fast reflexes, the vestibulo-ocular, vestibulo-spinal and vestibulo-collic reflexes which are essential for stabilising gaze and maintaining posture in dynamic conditions such as walking. Together with the visual^10^ and somatosensory systems, they form the multisensory sense of balance. In the case of abnormal vestibular function, visual and somatosensory inputs are not sufficiently precise and fast enough to efficiently substitute the impaired vestibular system, particularly in dynamic situations such as walking^11^, leading in an unsteady gait and oscillopsia^2^. These symptoms are even more pronounced in the dark or on uneven ground, when the visual or the somatosensory system can not operate optimally. One important consequence is an increased risk of falling^12^. Vestibular symptoms and their intensity can vary over time and from patient to patient^8^. Understanding the whole-body movements of patients with vestibulopathy could not only improve our knowledge of this pathology but also help in the development of new therapeutic solutions and provide clinicians with key elements to help them in their diagnosis.

Most research on the analysis of movement in patients with vestibulopathy has focused on spatiotemporal parameters^13–17^, which simply describe the general characteristics of the gait pattern (e.g. walking speed, step length, step time, etc.). The analysis of movement could also be based on kinematic parameters. Kinematics describe the way different body segments move in space^18^, enabling analysis of gait deviations in pathological groups compared to normal gait^19^. Indeed, kinematic curves enable detailed analysis of angular variations at the various joint or body segments concerned^19^. Furthermore, researchers have investigated either unilateral^11,13,14,17,20,21^ or bilateral^15,22^ vestibulopathy, but very few comparisons exist between these two groups^23^. We believe it is important to characterise and compare these pathological groups to obtain relevant outcomes for evaluating new treatments. To our knowledge, no study has analysed kinematic curves for the upper and lower limbs in UV and BV populations.

Moreover, several studies have shown that the dispersion of sample values and the variability of the data provide instructive information for a better understanding of the walking and balance difficulties encountered by vestibulopathy patients^14,15,24^. McCrum et al.^15^ suggested that the analysis of the variability of walking can be used as an assessment tool for vestibular interventions. It also seems relevant to us to explore this component to explain the movement of our patients. In other populations^25,26^, the gait standard deviation (GaitSD^27^) has been demonstrated to be a useful indicator of lower-body kinematic variability when only a small number of gait cycles are available, whereas more than 30 cycles are required for spatiotemporal parameters^27^. The GaitSD also condenses the variability of the participant’s gait into a single number. Although the literature lacks GaitSD data specific to patients with vestibulopathy, it is possible to draw initial conclusions by comparing them with data from the HS group.

Finally, clinical observations have reported stiffness of the upper limbs during walking in patients with vestibulopathy. In this sense, studies have shown that head and trunk movements^20,24,28,29^ are reliable indicators of how vestibulopathy affects patients’ movements. Head stabilisation appears as a crucial mechanism regulated by the vestibular system^28^, and is associated with the vestibulo-ocular reflex^30^ (VOR), which plays an essential role in stabilising gaze. Vestibulopathy patients cannot maintain their gaze on a fixed point during head movement, especially during a dynamic task, as the gaze shifts with the movement of the head away from the target^31–33^. This gaze instability causes oscillopsia and can lead to stiffness of the trunk to minimise symptoms. Pozzo et al.^28^ showed that BV patients have stiffness in the upper limbs and greater difficulty in stabilising the head in space. Moreover, as reported by Bronstein^34^ vestibulopathy patients showed impaired head stabilisation in space due to unpredictable body perturbations. To achieve a comprehensive analysis of the complete movements (i.e. kinematics) of vestibulopathy patients, we focused on examining the paticipants’ postural control during walking, with particular emphasis on head stabilisation. We therefore analysed the anchoring index (AI) of the head, as proposed by Assaiante and Amblard^35^. To our knowledge, few studies have looked at head movement in vestibulopathy patients^28,34^, and it could help to better understand the movements and problems experienced by these patients.

The present study aimed to investigate the body movement in BV and UV patients, using kinematics, gait variability, and head stabilisation, and to compare them with a group of healthy subjects (HS) during walking at three self-selected speeds: slow, comfortable, and fast. Characterising and understanding gait in these patients is a crucial step, both to improve the diagnosis and to help in the development of new treatments. To this end, a global kinematic study of segment movement analysis was first carried out and then used to focus successively on lower-body kinematic variability using the gait standard deviation (GaitSD)^27^ and on an upper-body parameter representing head stabilisation in space: the anchoring index (AI)^35^. Our first hypothesis was that BV and UV patients show some significant differences in kinematic patterns compared with HS (hypothesis 1). This hypothesis was put forward because strategies to compensate for vestibulopathy may have been acquired, as previously demonstrated in their spatiotemporal parameters^14,36^. Our second hypothesis was that variability during gait would be greater in BV and UV patients than in HS and greater during slow walking (hypothesis 2), which could explain the higher risk of falling^12^. Finally, the last hypothesis was that the patients' head stabilisation strategy would be a stabilisation in relation to the trunk rather than in relation to space, as an attempt to minimise disturbances caused by head movements within the body^28^ (hypothesis 3).

## Methods

### Participants

10 BV patients and 10 UV patients were recruited at the Division of Otorhinolaryngology and Head and Neck Surgery of a tertiary university hospital (Tables 1 and 2). No a priori power calculation was conducted, rather the number of participants was based on the available patients during the intended timeframe of the project. BV patients were diagnosed according to the guidelines of the Classification Committee of the Bárány Society^37^ (unsteadiness when walking or standing, oscillopsia and/or worsening of imbalance in darkness/uneven ground, no symptoms while sitting or lying down, bilaterally reduced or absent vestibulo-ocular reflex documented by a caloric test, video-head impulse test (vHIT), or torsion swing test, and finally not better accounted for by another disease). These strict inclusion criteria ensured that the patient population recruited corresponded to patients with severe bilateral vestibulopathy. UV patients had to meet clinical vHIT requirements, with gain values below 0.6 for the lateral semicircular canals of the affected ear. The UV had to be present for at least 3 months (to have a chronic deficit). Moreover, UV patients needed to have normal vestibular function in the other ear (vHIT gain values above 0.6). These strict criteria for UV and BV patients inclusion explained the difficulty of obtaining larger populations in pathological groups.

**Table 1:** Participant group characteristics.

	BV	UV	HS
n (female)	10 (5)	10 (5)	10 (6)
Age (years)	64.4 ± 9.6 (53.0; 82.0)	63.4 ± 6.2 (56.0; 77.0)	64.6 ± 10.0 (53.0; 82.0)
Height (cm)	1.64 ± 0.09 (1.51; 1.81)	1.67 ± 0.16 (1.47; 1.86)	1.71 ± 0.07 (1.63; 1.84)
Weight (kg)	71.4 ± 10.6 (53.0; 88.0)	79.6 ± 17.4 (54.0; 102.0)	70.9 ± 12.7 (56.0; 90.0)
BMI (kg/m^2^)	26.4 ± 3.8 (21.4; 34.2)	27.9 ± 2.4 (24.5; 31.0)	24.2 ± 3.6 (19.6; 29.1)
Analysed side	10 Left	4 Left, 6 Right	10 Left

Values are mean ± SD (min; max).

BV: bilateral vestibulopathy patients; UV: unilateral vestibulopathy patients; HS: healthy subjects.

**Table 2:** Aetiological data for vestibulopathy patients.

Patient	Sex	Affected side	Aetiology	Score DHI^38^
BV				
1	F	Both	Ototoxic	48
2	F	Both	Genetic	46
3	M	Both	Idiopathic	12
4	F	Both	Idiopathic	34
5	F	Both	Idiopathic	20
6	F	Both	Idiopathic	74
7	M	Both	Schwannoma	2
8	M	Both	Idiopathic	40
9	M	Both	Idiopathic	48
10	F	Both	Idiopathic	NA
UV				
1	F	L	Idiopathic	68
2	M	L	Idiopathic	6
3	M	L	Post-labyrinthectomy	64
4	F	R	Idiopathic	NA
5	M	L	Schwannoma	20
6	M	R	Idiopathic	8
7	F	R	Idiopathic	NA
8	M	R	Traumatic	11
9	F	R	Schwannoma	52
10	F	R	Idiopathic	14

BV: bilateral vestibulopathy patients; UV: unilateral vestibulopathy patients; Sex—F: female; M: male. NA: not available; L: left; R: right; DHI: Dizziness Handicap Inventory^38^.

Ten healthy participants were included in the HS group. They had no history of vestibular symptoms (i.e. imbalance, vertigo, dizziness) (Table 1). All HS met a criterion of normal vHIT gain values for all semicircular canals (vHIT gain values above 0.6).

All study participants were over 18 years of age and provided their written informed consent. The study was designed and conducted in accordance with the guidelines of the Declaration of Helsinki and was approved by the Cantonal Commission for Research Ethics of Geneva (NAC 11-080 CCER 11–219).

### Instruments and protocol

**Figure 1:**
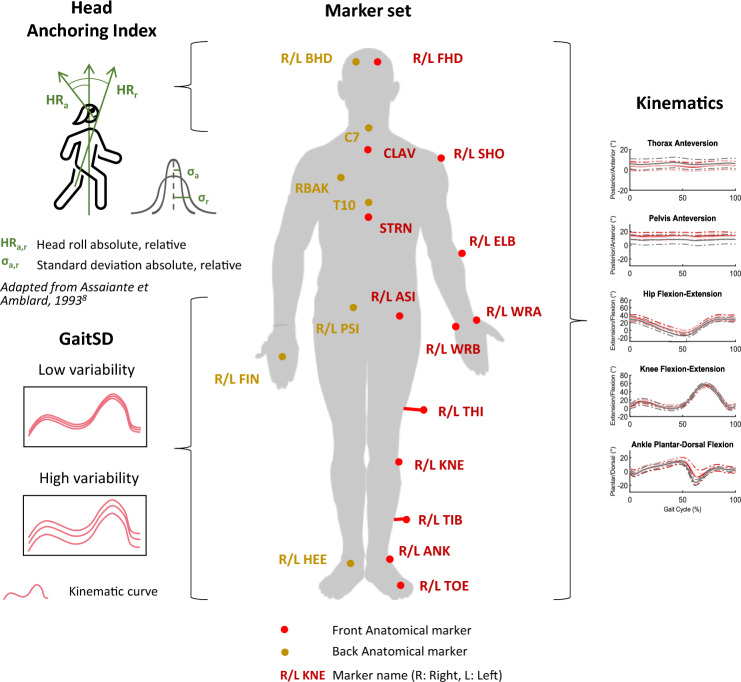
Summary of markerset and calculated variables. Red and yellow markers: Reflective markers placed according to the Conventional Gait Model 1.0.

A 12-camera motion capture system (Oqus 7+, Qualisys, Göteborg, Sweden), set at a 100 Hz sampling frequency, was used to track cutaneous reflective markers. The participants were equipped with 35 anatomical markers (14 mm diameter) placed on the whole body, according to the Conventional Gait Model 1.0^39,40^ (Fig. 1).

The gait analysis measurement started with a 10-s recording of the participant standing upright (T-pose). Then, participants were asked to walk barefoot back and forth on a 10-m walkway at three different self-selected speeds: slow, comfortable, and fast. Walking trials at each speed were repeated three times.

### Data processing

The marker trajectories were labeled using Qualisys Tracking Manager software (QTM 2019.3, Qualisys, Göteborg, Sweden) and exported in the C3D file format (https://www.c3d.org). All processing was performed using Matlab (R2021b, The MathWorks, Natick, MA, USA) with the C3D parser from the Biomechanics Toolkit (BTK)^41^. The marker trajectories were interpolated to fill gaps using a reconstruction method that relies on marker inter-correlations^42^. In each trial file, joint centers of the upper and lower limbs and the center of the posterior and anterior iliac spines were calculated and Ied as virtual markers. The hip joint centers were computed using Hara's regression equations^43^, while other joint centers were determined using a chord function^44^. Gait events, such as foot strikes and foot offs, were automatically detected using a custom-made algorithm developed in Matlab for self-selection among different methods^45^. To prevent detection errors, each event was visually verified by an operator.

### Data analysis

#### Kinematics

In this study, joint kinematics were calculated from the raw data of the marker trajectories (Fig. 1). A custom-made software developed by Moveck^®^ was used to compute the Conventional Gait Model 1.0^39,40^. Then, each trial was divided into gait cycles (foot strike to foot strike) to calculate all the parameters of this study. Kinematic angles were calculated for the three anatomical reference planes (sagittal, frontal, and transverse) and the joints and segments of the upper and lower limbs. Thus, angles for the head, neck, shoulder, elbow, wrist, trunk, spine, pelvis, hip, knee, ankle, and foot were calculated for each participant of the three studied groups (Fig. 1). The elbow ab-adduction, the elbow rotation, and the wrist rotation were not considered in the selection of trials because of their low angle amplitude. For lower limb angles, knee rotation, ankle ab-adduction, and ankle rotation were not included in the analysis either because of the low reliability of the calculations, in accordance with clinical gait analysis. A minimum of 2 cycles (due to missing data) was available per participant and condition. To normalise the weight for each group, all cycles of a single participant were averaged before averaging the values of each group. Preliminary analyses showed no significant difference between the left and right sides for BV and HS groups. Therefore, the left side of BV and HS groups was chosen for the analysis, and the affected side of UV patients. In summary, the 3 groups were compared using a set of twenty-five kinematic variables.

#### Gait Standard Deviation

The GaitSD was defined as the square root of the average variance of 9 kinematic variables in degrees^27^ (Formula 1: pelvis tilt, pelvis obliquity, pelvis rotation, hip flexion, hip abduction, rotation, knee flexion, ankle dorsiflexion, and foot progression angles) (Fig. 1). As for the kinematic curves, GaitSD was separated into the left side for the BV and HS groups and the affected side for the UV group. The calculation method^27^ strongly recommended selecting a minimum of 6 cycles to calculate a GaitSD with a relative precision superior to 90%. However, in the study, a minimum of 5 cycles per participant was chosen to avoid losing too many participants due to the low number of trials. Information on missing data can be found in a following section.

1 $${\text{GaitSD}} = \sqrt {\frac{1}{{\text{V}}}\sum\limits_{{{\text{k}} = 1}}^{{\text{V}}} {{\text{GVSD}}_{{\text{k}}}^{2} } }$$

where, V: number of kinematic variables; ${\text{GVSD}}_{{\text{k}}}^{2}$: gait variable standard deviation.

#### Anchoring Index

AI was calculated according to Schreiber et al.^46^ (Formula 2), based on the standard deviation of the head orientation in the global (laboratory) coordinate system and on the standard deviation of the head orientation relative to the trunk movement (Fig. 1). A positive AI value indicates a head stabilisation strategy in space (i.e. head and trunk move freely) and a negative value indicates a stabilisation strategy on the trunk^35^ (i.e. head and trunk move “in block”, head moves together with the trunk). As done in the GaitSD analysis, a minimum of 5 cycles per participant was needed to compute the AI. Information on missing data can be found in a following section.

2 $${\text{AI}} = \frac{{\upsigma _{{\text{r}}}^{2} - \upsigma _{{\text{a}}}^{2} }}{{\upsigma _{{\text{r}}}^{2} + \upsigma _{{\text{a}}}^{2} }}$$

where, σ_a_: standard deviation of the segment orientation in the global coordinate system (absolute); σ_r_: standard deviation of the segment in the trunk coordinate system (relative).

### Statistical analysis

Given the small number of participants in each study group (10 per group), we performed non-parametric statistical tests.

To study the differences between the kinematic curves of the three groups, a Statistical Parametric Mapping (SPM) (SPM1d, version 0.4.10) in Matlab using a non-parametric ANOVA was used. The Type I error rate was set to 0.05 (alpha) and the number of iterations, which is the number used to build the non-parametric distribution for the statistic test, was set to 100.

Differences in GaitSD and AI parameters between groups and for each walking speed were tested using a Kruskal–Wallis test. A post-hoc analysis was carried out to determine which groups showed a significant difference. A one-sample Wilcoxon test was also used to test if the AI was significantly different from zero, allowing us to determine whether the participants adopted specific or mixed strategies. In the case of mixed strategies, it means that stabilisation in space or in relation to the trunk can be found^46^. The significance threshold for the statistical tests was set at 0.05. Finally, the 95% confidence interval was calculated for the GaitSD and AI parameters from the median.

### Missing data

Due to the poor quality of the raw data before processing, only two trials (instead of three) were included in the analysis for the following participants and conditions (Table 3):

**Table 3:** Missing trials excluded from the full analysis.

Participants	Walking speed condition
BV_01	Fast
BV_02	Slow; Fast
BV_06	Slow
BV_10	Slow
UV_08	Slow
HS_09	Comfortable

Moreover, the following participant’s conditions were removed from the GaitSD and AI analyses because the number of cycles was less than 5 (Table 4):

**Table 4:** Missing trials excluded from the GaitSD and AI analyses.

Participants	Walking speed condition
BV_03	Fast
UV_02	Fast
UV_04	Fast
UV_10	Comfortable; Fast
HS_01	Comfortable; Fast
HS_03	Fast
HS_07	Fast
HS_09	Comfortable

## Results

The mean gait velocities measured for BV and UV patients and HS are presented in Table 5.

**Table 5:** Mean gait velocities for each group at each walking speed in m s^−1^.

Walking speed (m s^−1^)	BV	UV	HS
Slow walking speed	0.77 ± 0.14	0.72 ± 0.13	0.89 ± 0.12
Comfortable walking speed	1.04 ± 0.23	1.01 ± 0.19	1.29 ± 0.12
Fast Walking Speed	1.48 ± 0.27	1.57 ± 0.16	1.76 ± 0.15

Values are mean ± sd.

BV: Bilateral Vestibulopathy patients; UV: Unilateral Vestibulopathy patients; HS: Healthy subjects.

### Kinematics

**Figure 2:**
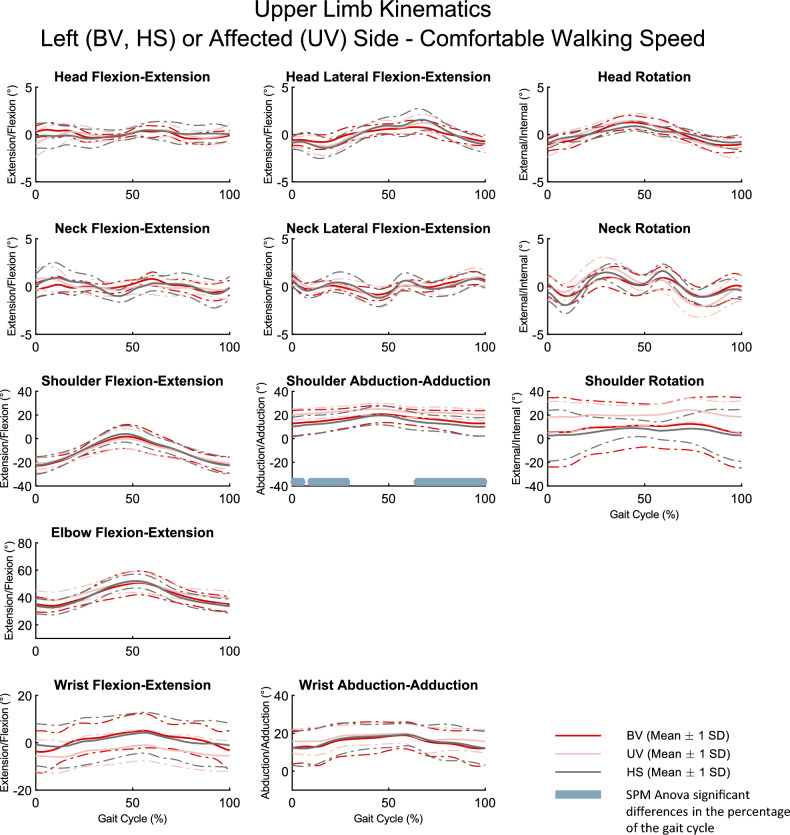
Upper limb kinematic curves for the left or affected side, respectively BV, HS, and UV groups, at comfortable walking speed. Solid lines : mean kinematic curve for each group. Dotted lines: standard deviation (SD) kinematic curve for each group. Grey bar: significant SPM results (ANOVA) as a percentage of the gait cycle. BV: bilateral vestibulopathy patients; UV: unilateral vestibulopathy patients; HS: Healthy subjects.

**Figure 3:**
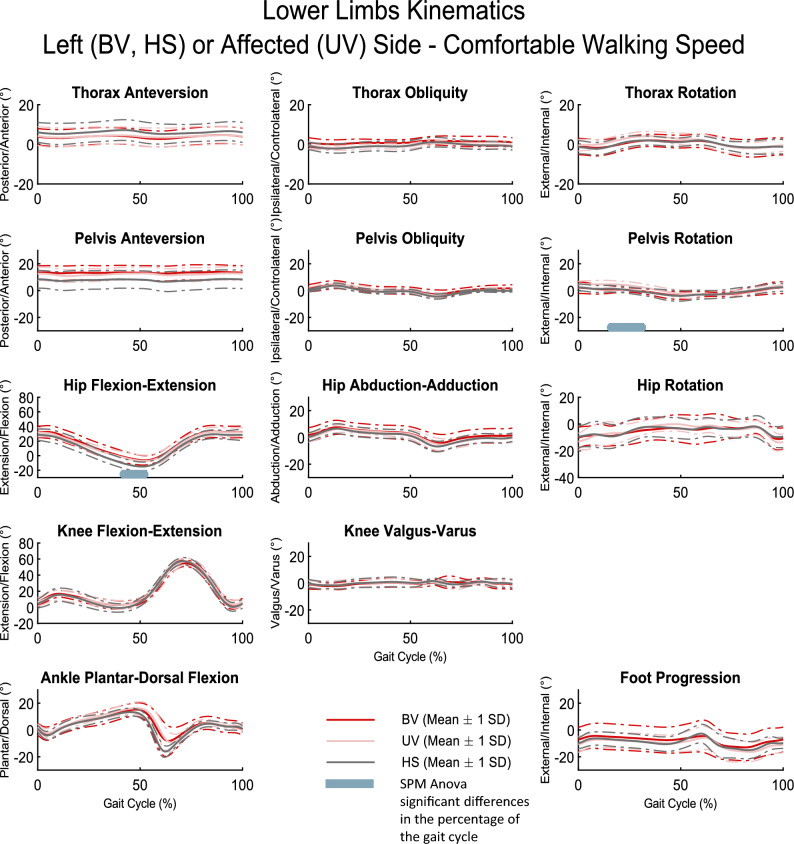
Lower limb kinematic curves for the left or affected side, respectively BV, HS, and UV groups, at comfortable walking speed. Solid lines : Mean kinematic curve for each group. Dotted lines: Standard deviation (SD) kinematic curve for each group. Grey bar: significant SPM results (ANOVA) as a percentage of the gait cycle. BV: bilateral vestibulopathy patients; UV: unilateral vestibulopathy patients; HS: Healthy subjects.

Figures 2 and 3 show the mean of the kinematic curves and standard deviation for the three groups at comfortable walking speed. Overall, the results were visually similar between the pathological (BV and UV) groups and the HS group. Kinematic curves presented almost superimposed patterns between each of the groups. This visual comparison was consolidated by the SPM analysis, which revealed significant differences (p < 0.05) on only a few kinematic parameters between the three groups: during shoulder abduction–adduction (0–5.6%; 8.5–29.1%; 64.0–100% of gait cycle), pelvis rotation (14.7–31.7% of gait cycle), and hip flexion–extension (41.0–52.6% of gait cycle) (see bars in Figs. 2 and 3). Similar results were obtained for the two other walking speeds (slow and fast) that are available in Supplementary File 1.

### Gait Standard Deviation

**Figure 4:**
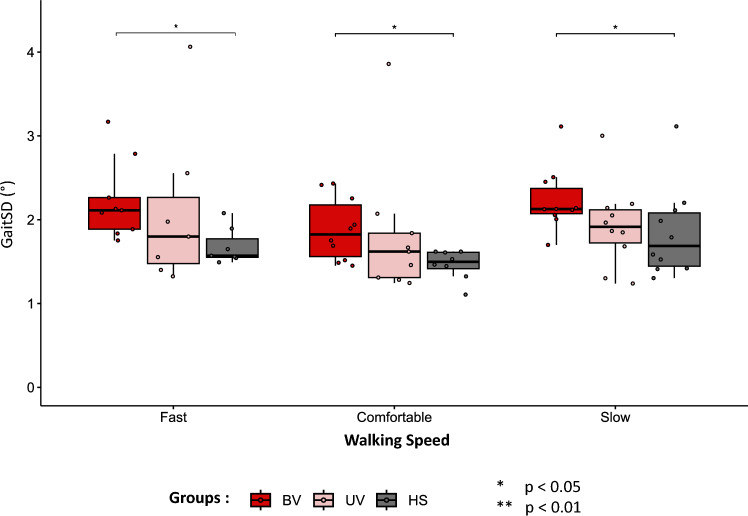
Gait Standard Deviation (GaitSD) in degrees (°) for each group at each walking speed condition for the left side (BV, HS) or affected side (UV). Box plots indicate Median values, Q1 and Q3 percentile values (colored box) as well as min and max percentile values (error bars). A point corresponds to the individual results of one subject. * p < 0.05, ** p < 0.01. BV: bilateral vestibulopathy patients; UV: unilateral vestibulopathy patients; HS: Healthy subjects.

Results for the left side (BV, HS) or affected side (UV) are presented in Fig. 4. Results of the Kruskal–Wallis test and the post-hoc test showed that the GaitSD was significantly superior only for the BV group compared to the HS group at each walking speed condition. The median GaitSD value of the BV group at comfortable walking speed was 1.82° (95%CI [1.59–2.06]) whereas for the HS group, it was at 1.50° (95%CI [1.14–1.85]) (p = 0.018). Similar significant results were observed for the two other walking speeds between BV and HS (fast walking speed: p = 0.018; slow walking speed: p = 0.035). Additionally, within-group comparisons showed that GaitSD was not significantly higher at slow and fast walking speeds compared to comfortable speed. For example, medians of 2.13° and 2.12° were found at slow and fast speeds respectively, while a median of 1.82° was found at comfortable speed for the BV group. Likewise, the other two groups demonstrated similar results. The results are presented by groups and with 95% confidence intervals in Supplementary File 2.

### Anchoring Index

**Figure 5:**
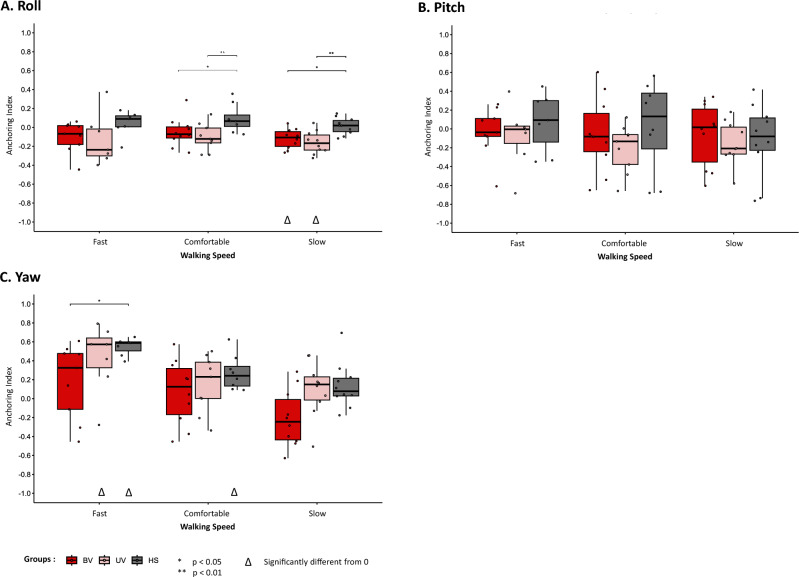
Anchoring Index (AI) as a function of walking speed for all subjects for (**A**) roll, (**B**) pitch, and (**C**) yaw. Box plots indicate Median values, Q1 and Q3 percentile values (colored box) as well as min and max percentile values (error bars). A point corresponds to the individual results of one subject. *p < 0.05, **p < 0.01. Δ: significant difference from 0 (p < 0.05). BV: bilateral vestibulopathy patients; UV: unilateral vestibulopathy patients; HS: healthy subjects.

AI results are shown in Fig. 5. The Kruskal–Wallis test and the post-hoc test showed significant differences between the vestibulopathy groups and the HS group for the roll movement (Fig. 5A). AI was significantly lower for the BV group compared to the HS group (p = 0.017 at slow walking speed, p = 0.042 at comfortable walking speed), as well as for the UV group compared to the HS group (p = 0.003 at slow walking speed, and p = 0.008 at comfortable walking speed). No significant differences were found for the pitch movement (Fig. 5B). Concerning the significant differences for the yaw movement (Fig. 5C), the statistical test showed differences only between the BV group and the HS group at slow walking speed (p = 0.031).

Moreover, the conditions for which the AI values were significantly different from zero were few: BV (p = 0.010, r = 0.79) and UV (p = 0.006, r = 0.82) groups at slow speed for roll angle, UV (p = 0.047, r = 0.77) and HS (p = 0.016, r = 0.89) groups at fast speed for yaw angle, and HS (p = 0.008, r = 0.89) at comfortable speed for yaw angle. This means that participants adopted few specific strategies (stabilisation in the trunk or in space), but that mixed strategies were adopted. Stabilisation in space or in the trunk could thus be found.

Furthermore, there was greater variability in the data for pitch and yaw angles. This was confirmed by the 95%CI analysis (Supplementary File 3), where the intervals were smaller for the roll angle. AI values for the HS group were generally above zero, while for the pathological groups (BV and UV), AI values fluctuated between negative and positive values depending on conditions and angles. Yet most median values were close to zero.

## Discussion

This study aimed to investigate global movement patterns in patients with chronic vestibulopathy (unilateral and bilateral), compared to a group of healthy subjects. Our main results showed only a few significant differences between groups in terms of kinematic joint movements, rejecting hypothesis 1, according to which UV and BV patients differ from HS. Our hypothesis 2 was that variability during walking would be greater in UV and BV patients. This was confirmed for the BV group but rejected for the UV group. Regarding the AI, significant differences appeared between BV and HS groups for the roll and pitch movements, but not for the yaw movements. The only significant difference in AI for the UV group corresponded to the roll movement at a slow speed. This partially confirms hypothesis 3, in which the patient’s head stabilisation strategy would be stabilisation in relation to the trunk rather than in relation to space.

### Kinematics

At a comfortable walking speed, the kinematic curves of the BV and UV groups closely resemble those of the HS group. This similarity can be explained by the fact that motor functions are not affected in patients with chronic vestibulopathy. Neurological impairments generally fall into two categories : those affecting motor functions, such as the motor disorders of cerebral palsy^47^ or stroke^48^, which result in spasticity, muscle weakness, or muscle retractions that hamper walking. And those involving sensory impairments such as blindness^49^, neuropathies (diabetes^50^), or vestibulathy, which have less influence on walking. Motor functions are not directly affected, and only the damaged senses result in gait patterns that differ from those of healthy subjects. Vestibulopathy leads to balance disorders but does not directly affect motor functions of the lower limbs. This may explain why few significant differences were found in this study for patients with vestibulopathy. Moreover, patients with chronic vestibulopathy have most likely acquired strategies that enable them to compensate for their gait deficits, notably through daily training and physiotherapy. This would explain why their gait differs little from that of the control group^51^. The only significant differences observed could be explained by a search for stability. For example, the shoulder adduction caused the arms to spread, potentially increasing balance^52^. For hip extension, patients with a vestibulopathy walk with smaller steps, which requires less hip amplitude and therefore less hip extension, hence the few differences observed in the curves. In terms of pelvis rotation, patients show greater rotation than healthy subjects. This increase in the pelvis rotation could be explained by compensation for the lack of hip amplitude in the sagittal plane to preserve step length^53^. However, the most important message to retain is that the differences are significant but remain small. We can not really target any specific orthopedic interventions, but perhaps more recommendations for physiotherapy sessions, to work on these parameters. Furthermore, it should be mentioned that walking can be considered a "fairly easy" task to perform for patients compared to more discriminative tasks such as walking on uneven ground^37^ or walking in the dark^37,54^. Therefore, adding a perturbation such as a support base perturbation could still reveal important differences^22^.

### Gait Standard Deviation

Several studies have demonstrated the influence of vestibulopathy on gait variability^14,15,24^. These results were observed in particular in the study of McCrum et al.^15^ which showed that gait variability could be used for the characterisation of the gait of vestibulopathy patients. Comparison of gait variability (GaitSD) between groups revealed significant differences between the BV and HS groups at each walking speed, consistent with clinical observations. Indeed, BV patients tend to deviate from a linear path^55^ and lose balance but can catch up thanks to compensation from other sensory inputs such as visual^56^ or proprioceptive^57^ inputs.

It was difficult to compare GaitSD values with the literature, as the majority of studies have focused on this parameter in other populations. Tabard-Fougère et al.^25^ showed that in children and young adults with cerebral palsy, GaitSD values were similar to those obtained in this study (2.5 ± 0.8° for unilateral cerebral palsy children ; 2.5 ± 0.8° for bilateral cerebral palsy children). However, a precise comparison of these values is inappropriate due to the difference in pathologies and populations.

Interestingly, although previous studies have demonstrated that walking speed can significantly affect kinematic^58^ and spatiotemporal parameters^15^, this did not appear to influence the parameters investigated. In this study, the impact of walking speed had low discriminating power between groups. In particular, fast walking did not seem to have a big impact on pathological groups, whereas the slow walking task seems to be difficult in patients^11^. In this study, the self-selected slow walking speed was not as slow as some of the slow walking speeds imposed in other studies^14,15,23^. It should be also noted that participants (see Missing data section) had to be excluded from the GaitSD calculation because the number of cycles was insufficient to be included in the analysis. Below 5 gait cycles, the GaitSD calculation is not stable^27^.

### Anchoring Index

The AI defined by Assaiante et Amblard^35^ was used to describe head stabilisation strategies during walking. Overall, results show that patients with vestibulopathy tend to stabilise their head relative to the trunk whereas healthy subjects tend to stabilise their head in space. This means that during walking the patient’s movement was “in block”, e.g. when the patient turned his head to the right, the trunk also turned^28,59^. This was only true for roll movements, with negative anchoring index values, especially at comfortable and fast walking speeds. This is consistent with the work of Pozzo et al.^28^, who showed that patients with vestibulopathy have upper body stiffness (head/trunk rigidity). The low AI values obtained for the roll movement can be explained by a small head movement in this plane. For the other planes (pitch and yaw), the patients with vestibulopathy showed AI values that decreased according to walking speed, similar to the HS group. Thus, patients might adopt different stabilisation strategies depending on walking speed and head movement. The head seems to be more stabilised in space at higher speeds, whereas stabilisation seems to be more relative to the trunk at lower speeds. This is consistent with our results, even for HS. Particularly for pitch, BV and UV patients will preferentially adopt a strategy of stabilising the head on the trunk (values close to zero or negative). In contrast, for yaw, all the groups will be more likely to stabilise their head in space (values close to zero or positive). Nevertheless, these values decreased with slower speeds. Values obtained for HS in this study were consistent with those reported by Assaiante and Amblard^35^. Head stabilisation in space is naturally achieved by HS. It is interesting to note that the variability of the data is high, particularly for the HS group. It can therefore probably be concluded that patients and subjects adopted different head stabilisation strategies depending on the conditions.

### Study limitations

The main limitation of this study is the low number of participants in each group (10) and the low number of trials per condition. As for the small number of participants, the prevalence of this pathology is low and we wanted to respect the selection criteria of the Bárány Society^37^ to ensure that we made a correct diagnosis of the pathology, and to characterise severe vestibulopathy as accurately as possible. Less selective criteria would have made it possible to have a larger cohort. It should also be noted that other studies in the field have even smaller sample size^14,16,23^. Therefore, we are convinced that this dataset is valid and provides a unique starting reference for future studies on larger cohorts.

Even though there was no a priori sample size calculation, a posteriori power calculation (G*Power: Statistical Power Analyses software) was carried out with the differences found in the head AI roll movement at comfortable walking speed between the 3 groups (-0.05 ± 0.16 for BV; -0.10 ± 0.14 for UV; 0.09 ± 0.15 for HS). The power obtained with a post hoc test was 0.70 (effect size: f = 0.54; α = 0.05; sample size: n = 30; 3 groups). To achieve a satisfactory statistical power of 0.80, the sample size calculation (G*Power: Statistical Power Analyses software) for the estimation (power = 0.80; α = 0.05) of a difference between the 3 groups based on a one-way ANOVA required a total sample size of 39 participants. A minimum of 13 participants should therefore be recruited per group. In conclusion, we are not so far from the required number of participants, but for a greater statistical power (power = 0.95; α = 0.05) a total sample size of 60 would be required.

The low number of gait trials per condition can be explained by the duration of the measurement, which should not exceed an hour to avoid excessive patient fatigue. We are aware that these factors may create limitations to this study, and we encourage any other study to validate our results on larger sample sizes.

We also wanted the gait to be as natural as possible, so walking speeds were chosen by participants according to their understanding of the instructions. Spontaneous speeds were therefore not as identical from subject to subject, group to group, and trial to trial as when walking on treadmills^15^. It is also interesting to note that other studies^14,36,60^ on the same populations do not obtain self-selected walking speeds with such large differences between groups (for example, 1.00 ± 0.18 m s^−1^ for patients with bilateral vestibulopathy against 1.11 ± 0.19 m s^−1^ for healthy subjects^60^), probably due to the data acquisition conditions (i.e. walking on a treadmill).

Regarding the presence of outliers in the HS group, they were only screened for vestibular deficits but not for other pathologies that could impact gait patterns. It should be noted that none of the subjects reported any known pathology. Non-parametric statistical tests also reduced the influence of outliers in the analysis of the results.

In addition, the BV group included physically active patients, some of whom were also undergoing physical therapy. This could have created a bias in our selection. Being physically active can reduce the impact of vestibulopathy on simple walking tasks^61^ as those used in this study. However, no data collection (i.e. questionnaires) was done to quantify patient’s activity or whether they consulted a physiotherapist. This information was relied on from patients’ comments. Since it was not structured within the study, the information was not considered in the analyses, which may constitute a further limitation of the study.

Finally, this study presented the results of patients tested in standard walking conditions, in which patients were not challenged to their maximum capacities. During simple walking tasks such as those used in this study, head movement can be minimised which also reduces vestibular input. Furthermore, as stated above, patients suffer from chronic, long-duration pathology and might have acquired compensation strategies for getting around in everyday life. The DHI scores (Table 2) reflect differences in symptoms between patients but have not been analysed in more detail in this study. More discriminative tasks should be performed to classify the groups more precisely. It was previously reported that walking in the dark^3^ or walking on a narrow base^62^ are the most difficult tasks for these populations. Future studies should therefore focus on analysing these tasks. Analyses of every day “complex” tasks (putting on shoes, getting on a bus, walking while reading signs, etc.) would also provide a better understanding of what limits patients.

## Conclusion

This study showed that the full-body kinematic curves obtained were mostly similar between the vestibulopathy patients and healthy subjects. Gait variability was significantly higher for the BV group compared to the HS group at each walking speed but no difference was observed for the UV group compared to the HS group. Head stabilisation strategies were different depending on the conditions, BV and UV groups tended to stabilise their head in relation to their trunk whereas the HS group tended to stabilise their head in space during the roll movement. Characterisation of groups with vestibulopathy was limited using tests in standard walking conditions, because of compensation of other visual and proprioceptive inputs. The specific parameters studied (GaitSD, AI) appear as relevant parameters for vestibular assessment.

## Supplementary Information

Supplementary Information.

## Data Availability

Raw data in C3D format are available in Zenodo repository, doi : https://zenodo.org/record/8210552. It is possible to read C3D files with the open-source software Mokka (http://biomechanical-toolkit.github.io/). Kinematic, GaitSD, and AI results are available in CSV file format in Zenodo repository, doi : https://zenodo.org/record/8210552.
