# Author Correction: Full‑body kinematics and head stabilisation strategies during walking in patients with chronic unilateral and bilateral vestibulopathy

**DOI:** 10.1038/s41598-024-64174-6

**Published:** 2024-06-11

**Authors:** Gautier Grouvel, Anissa Boutabla, Julie Corre, Rebecca Revol, Marys Franco Carvalho, Samuel Cavuscens, Maurizio Ranieri, Jean-François Cugnot, Christopher McCrum, Raymond van de Berg, Nils Guinand, Angélica Pérez Fornos, Stéphane Armand

**Affiliations:** 1 Division of Otorhinolaryngology Head and Neck Surgery, Geneva University Hospitals and University of Geneva, Geneva, Switzerland; 2 Kinesiology Laboratory, Geneva University Hospitals and University of Geneva, Geneva, Switzerland; 3 Division of Clinical Neurosciences, Geneva University Hospitals, Geneva, Switzerland; 4 Department of Nutrition and Movement Sciences, NUTRIM School of Nutrition and Translational Research in Metabolism, Maastricht University Medical Center+, Maastricht, The Netherlands; 5 Division of Balance Disorders, Department of Otorhinolaryngology and Head and Neck Surgery, Maastricht University Medical Center+, Maastricht, The Netherlands

Correction to: *Scientific Reports* 10.1038/s41598-024-62335-1, published online 23 May 2024

The original version of this Article contained an error in Reference 46, which was incorrectly given as:

Schreiber, C., Remacle, A., Chantraine, F., Kolanowski, E. & Moissenet, F. Influence of a rhythmic auditory stimulation on asymptomatic gait. *Gait Posture* **50**, 17–22 (2016).

The correct reference is listed below:

Schreiber, C. *et al*. Influence of walking velocity on strategies of head stabilisation. *Mov. Sport Sci. Sci. Motric.*, **93**, 57–61 (2016).

The original Article has been corrected.

