# Author Correction: Full-body kinematics and head stabilisation strategies during walking in patients with chronic unilateral and bilateral vestibulopathy

**DOI:** 10.1038/s41598-024-74552-9

**Published:** 2024-10-04

**Authors:** Gautier Grouvel, Anissa Boutabla, Julie Corre, Rebecca Revol, Marys Franco Carvalho, Samuel Cavuscens, Maurizio Ranieri, Jean‑François Cugnot, Christopher McCrum, Raymond van de Berg, Nils Guinand, Angélica Pérez Fornos, Stéphane Armand

**Affiliations:** 1 Division of Otorhinolaryngology Head and Neck Surgery, Geneva University Hospitals and University of Geneva, Geneva, Switzerland; 2 Kinesiology Laboratory, Geneva University Hospitals and University of Geneva, Geneva, Switzerland; 3 Division of Clinical Neurosciences, Geneva University Hospitals, Geneva, Switzerland; 4 Department of Nutrition and Movement Sciences, NUTRIM School of Nutrition and Translational Research in Metabolism, Maastricht University Medical Center+, Maastricht, The Netherlands; 5 Division of Balance Disorders, Department of Otorhinolaryngology and Head and Neck Surgery, Maastricht University Medical Center+, Maastricht, The Netherlands

Correction to: *Scientific Reports*10.1038/s41598-024-62335-1, published online 23 May 2024

The Funding section in the original version of this Article was omitted. The Funding section now reads:

“The authors declare financial support was received for the research, authorship, and/or publication of this article. This work was supported by the Swiss National Science Foundation (BRIDGE Nos. 40B2-0_20356), which do not necessarily endorse opinions, interpretations, and conclusions of the authors. The funders were not involved in the study design, collection, analysis, interpretation of data, the writing of this article, or the decision to submit it for publication.”

The original Article has been corrected.

