# Supplementary material for: Full-body kinematics and head stabilisation strategies during walking in patients with chronic unilateral and bilateral vestibulopathy
Source: Sci Rep. 2024 May 23;14:11757. doi: 10.1038/s41598-024-62335-1 (PMC11116555; doi:10.1038/s41598-024-62335-1)

# Supplementary data

### Title

Full-body kinematics and head stabilisation strategies during walking in patients with chronic unilateral and bilateral vestibulopathy

### Authors

Gautier Grouvel^1,2^, Anissa Boutabla^1^, Julie Corre^1^, Rebecca Revol^1^, Marys Franco Carvalho^2^, Samuel Cavuscens^1^, Maurizio Ranieri^1^, Jean-François Cugnot^3^, Christopher McCrum^4^, Raymond van de Berg^5^, Nils Guinand^1^, Angelica Perez-Fornos^1^, Stéphane Armand^2^

**Affiliations**

1. Division of Otorhinolaryngology Head and Neck Surgery, Geneva University Hospitals and University of Geneva, Geneva, Switzerland

2. Kinesiology Laboratory, Geneva University Hospitals and University of Geneva, Geneva, Switzerland

3. Clinical Neurosciences Department, Neurorehabilitation Department, Geneva University Hospitals, Geneva, Switzerland

4. Department of Nutrition and Movement Sciences, NUTRIM School of Nutrition and Translational Research in Metabolism, Maastricht University Medical Center+, Maastricht, The Netherlands

5. Division of Balance Disorders, Department of Otorhinolaryngology and Head and Neck Surgery, Maastricht University Medical Center+, Maastricht, The Netherlands

corresponding author(s): Gautier Grouvel (gautier.grouvel@unige.ch)

### Supplementary data

**Supplementary File 1** – (A) Upper limb kinematic curves for the left (BV, HS) or affected side (UV) at slow walking speed. (B) Lower limb kinematic curves for the left (BV, HS) or affected side (UV) at slow walking speed. (C) Upper limb kinematic curves for the left (BV, HS) or affected side (UV) at fast walking speed. (D) Lower limb kinematic curves for the left (BV, HS) or affected side (UV) at fast walking speed. Solid lines : Mean kinematic curve for each group. Dotted lines : Standard deviation (SD) kinematic curve for each group. Grey bar: significant SPM results (ANOVA) as a percentage of the gait cycle. BV: Bilateral vestibulopathy patients; UV: Unilateral vestibulopathy patients; HS: Healthy subjects.

**
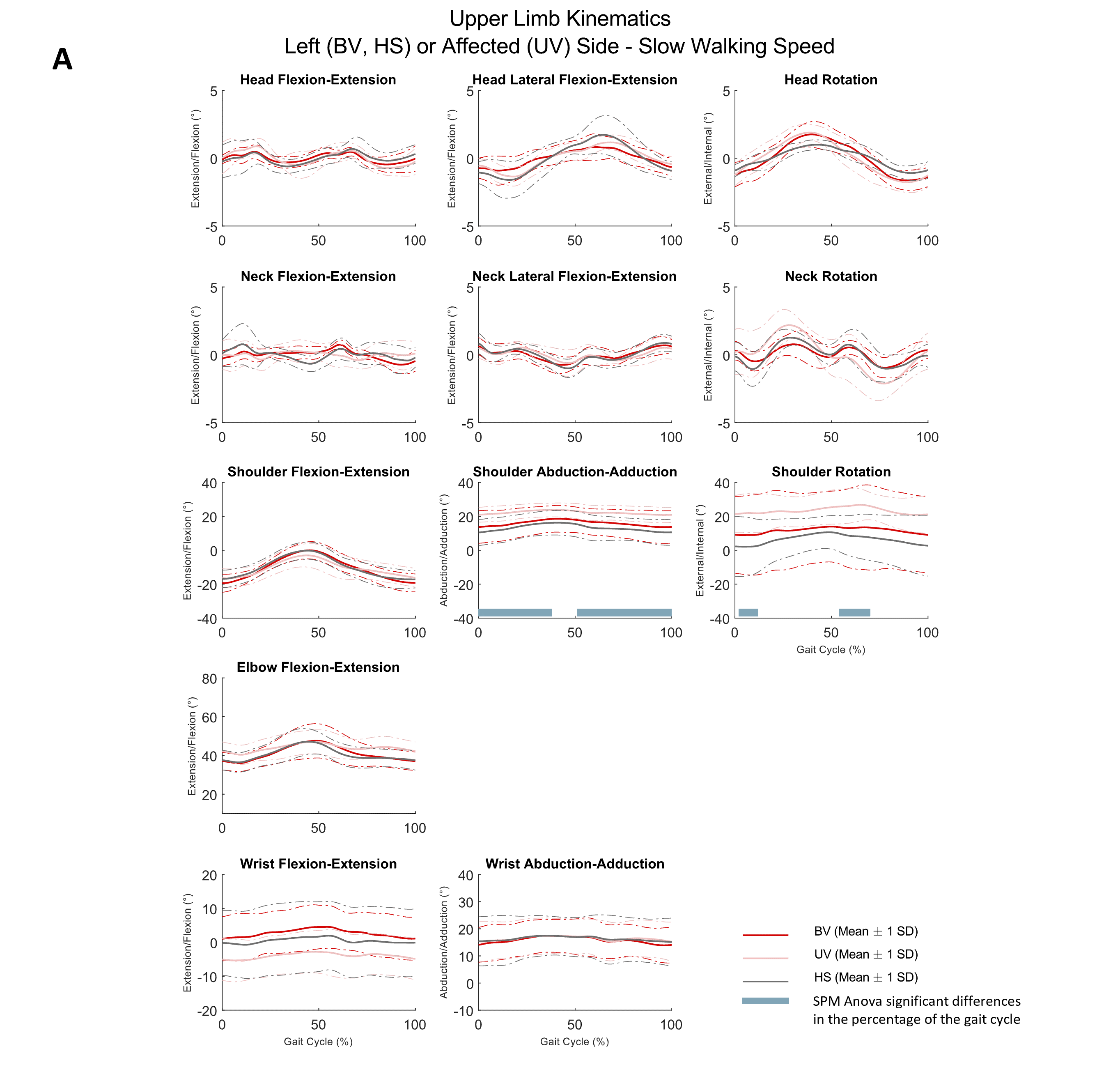
**

**
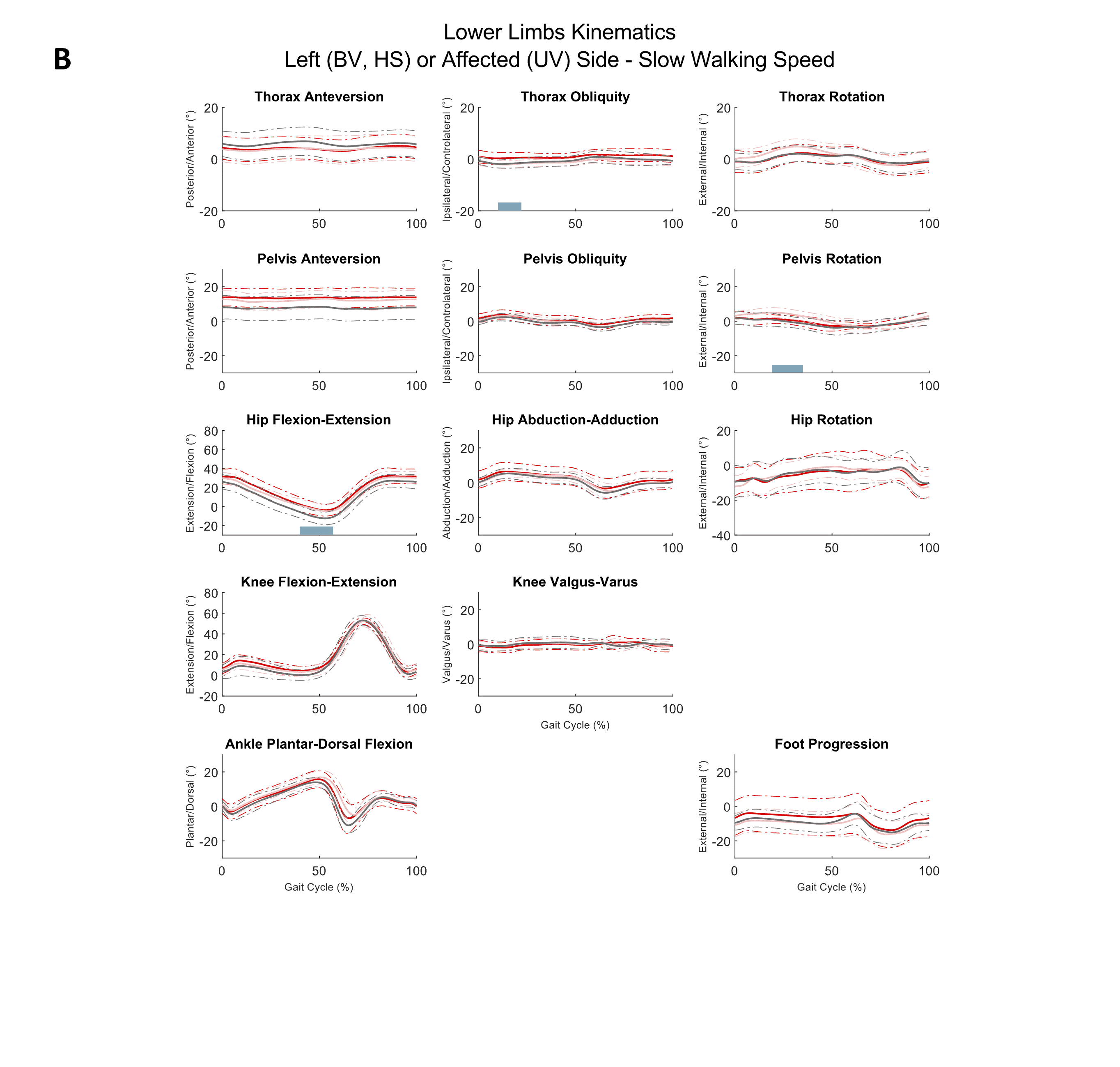
**

**
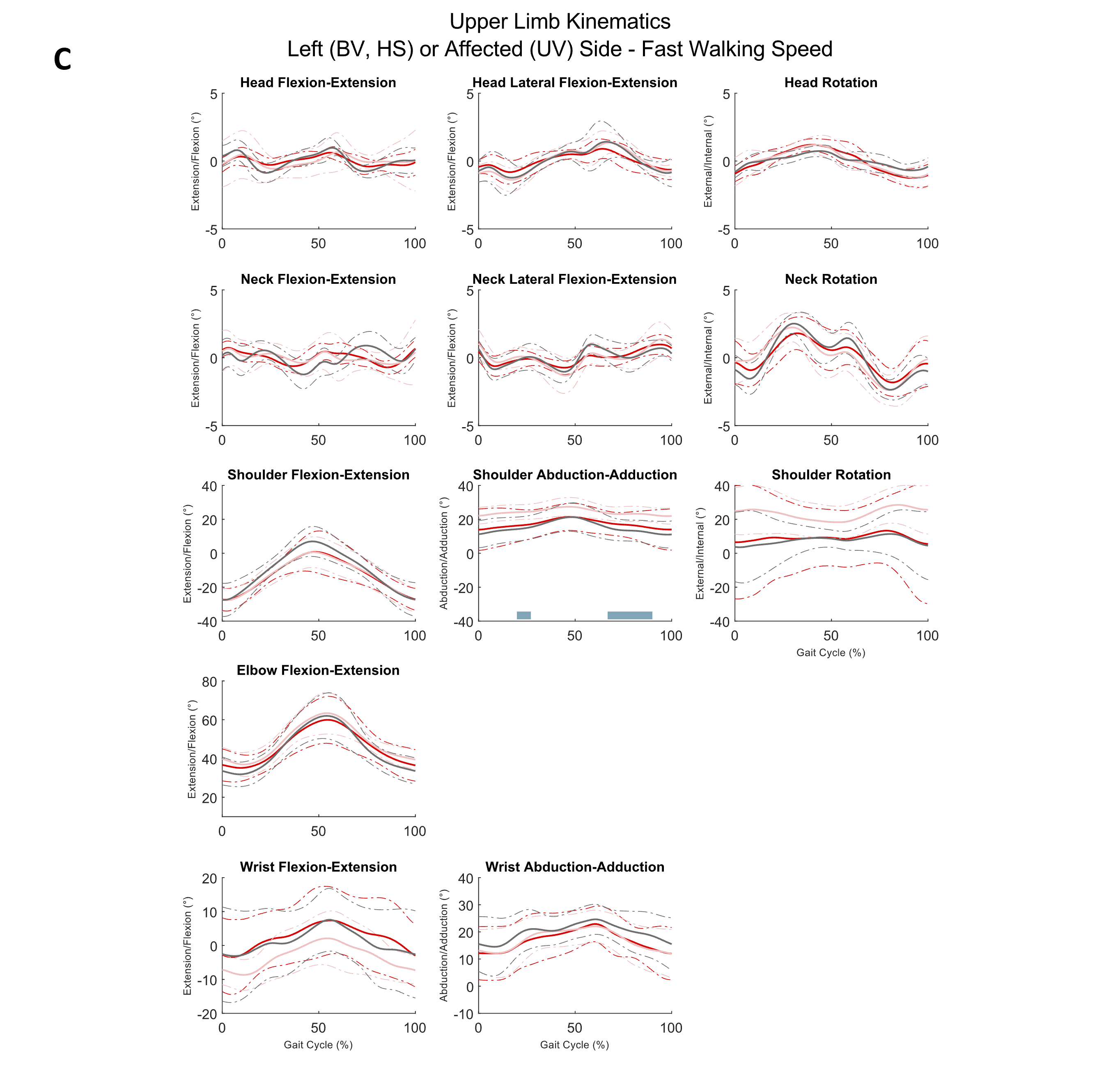
**

**
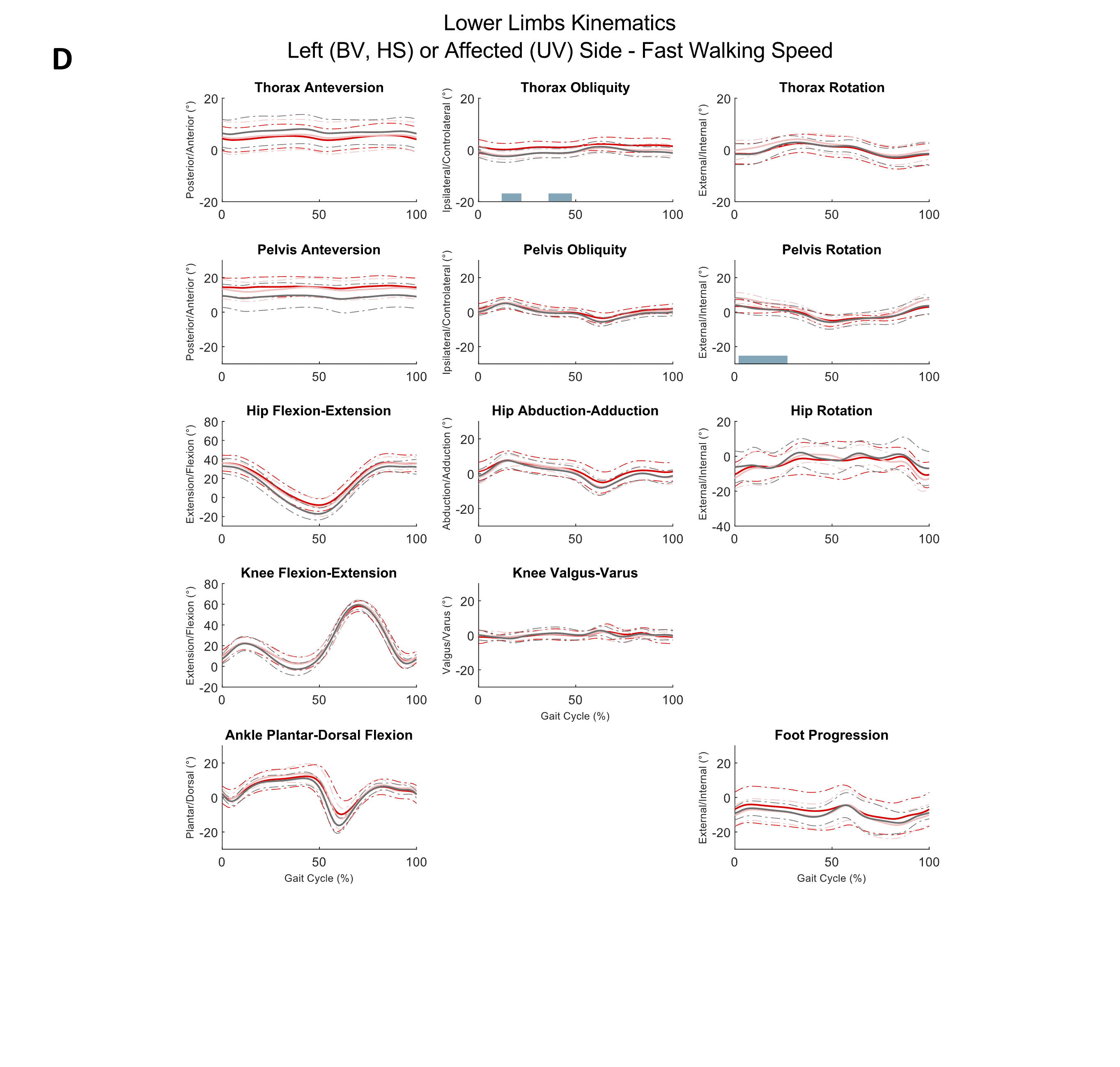
**

**Supplementary File 2** – 95 % confidence intervals of Gait Standard Deviation (GaitSD) in degrees for each group at each walking speed condition. Middle point corresponds to median value. Dotted lines correspond to minimum and maximum values of 95% confidence intervals for the HS group for all walking speeds. BV: Bilateral vestibular patients; UV: Unilateral vestibular patients; HS: Healthy subjects.


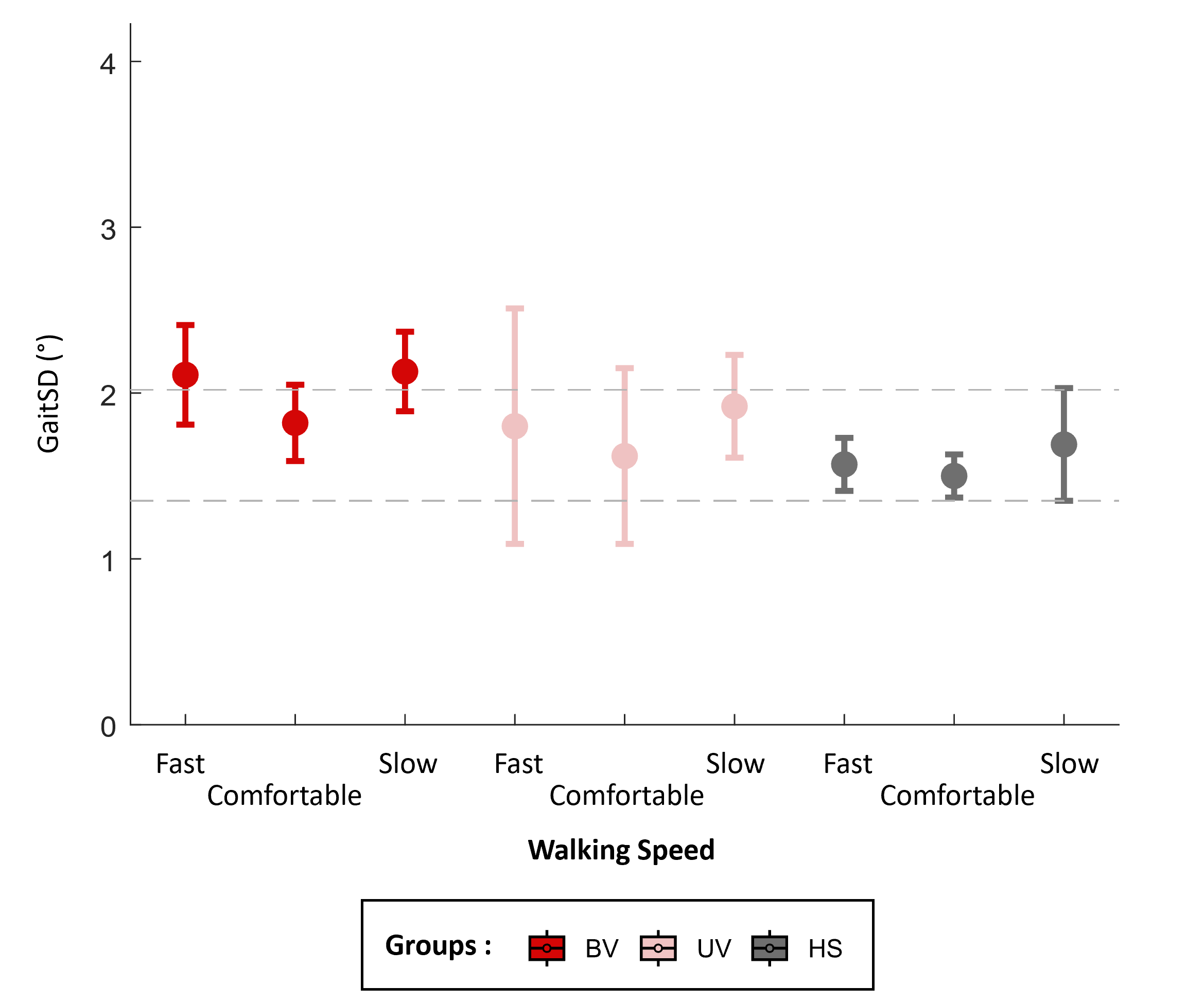


**Supplementary File 3** – 95 % confidence intervals of Anchoring Index (AI) as a function of walking speed for all subjects for (A) roll, (B) pitch, and (C) yaw. Middle point corresponds to median value. BV: Bilateral vestibulopathy patients; UV: Unilateral vestibulopathy patients; HS: Healthy subjects.


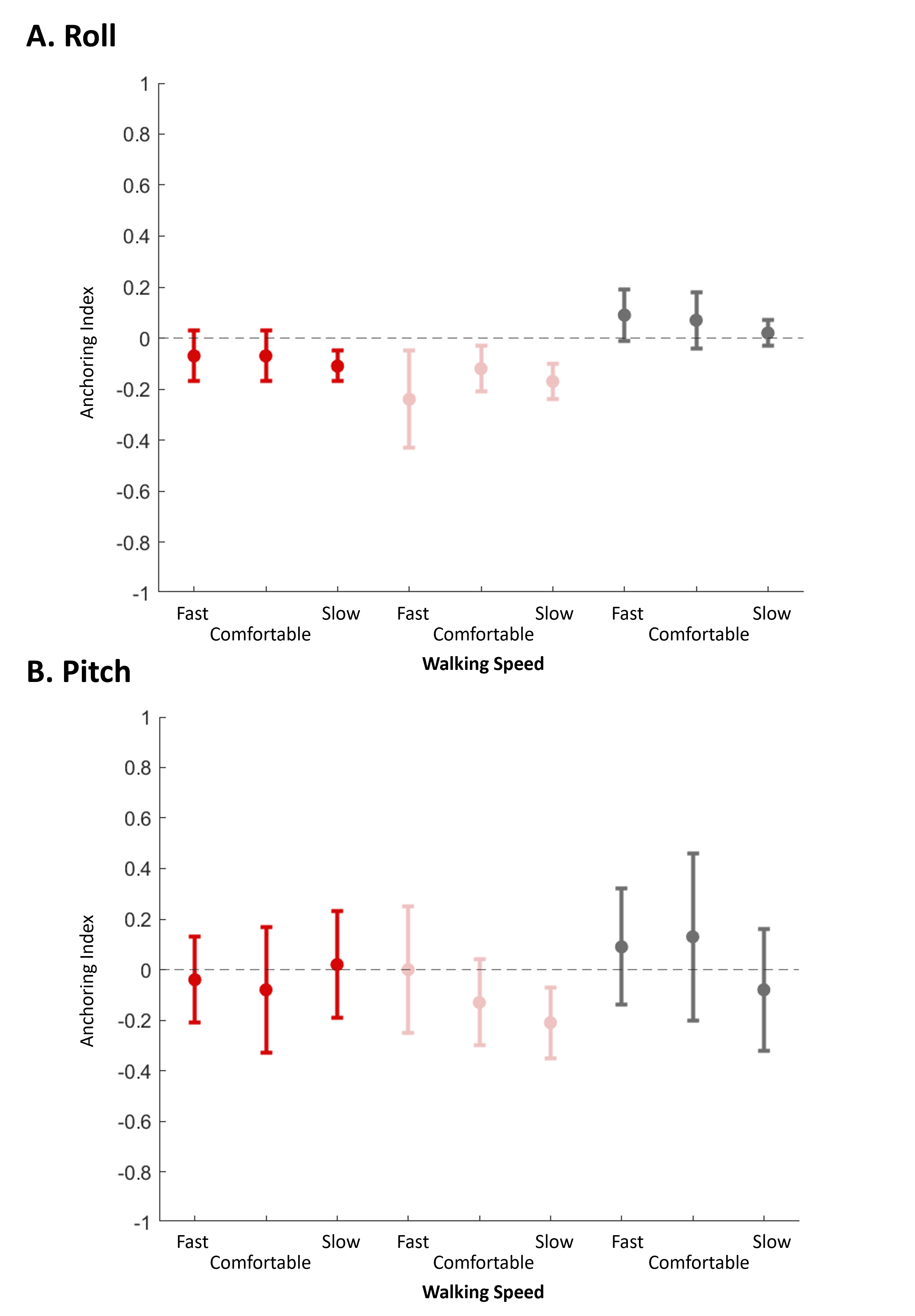


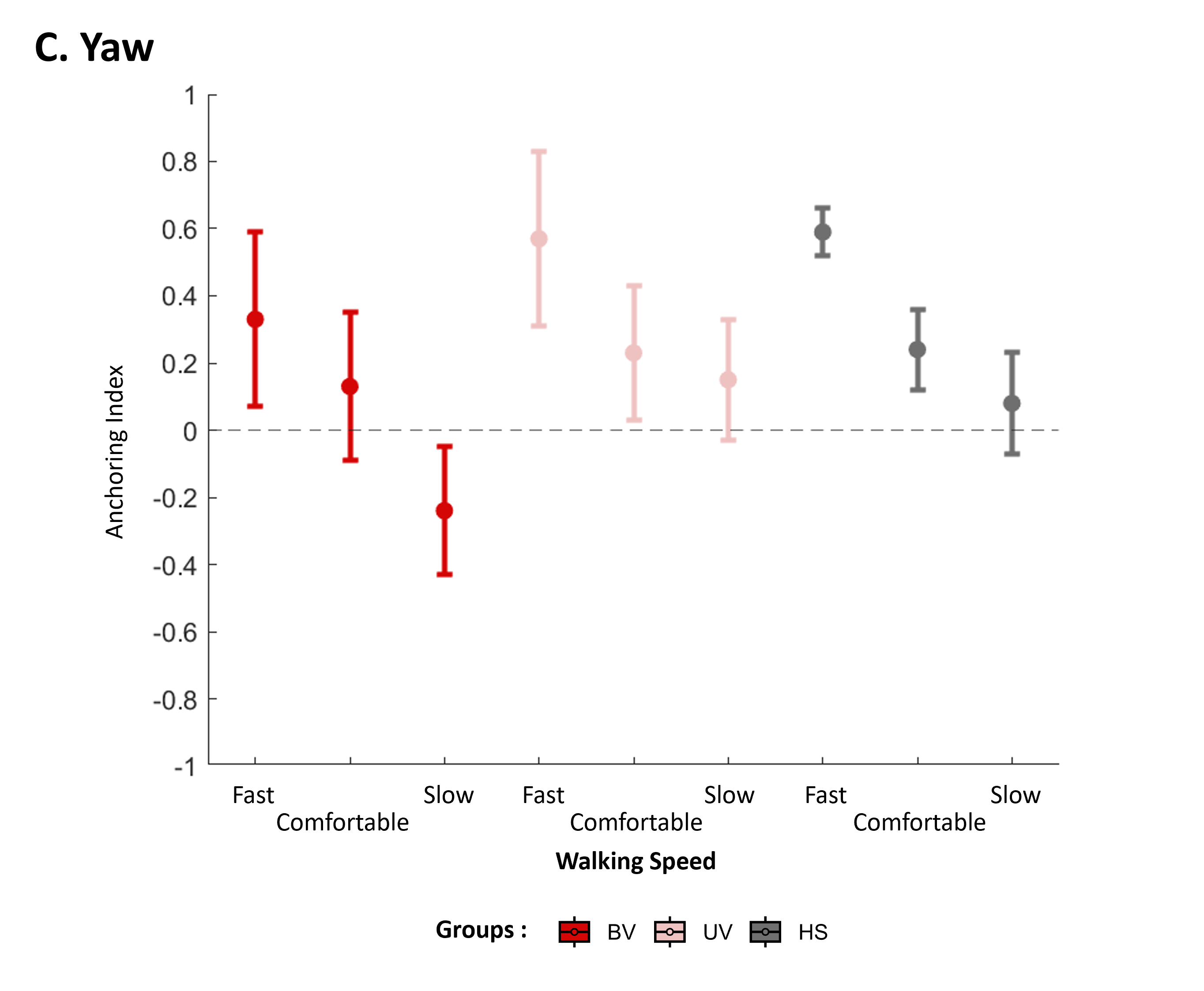

Supplement: Supplementary file 1 — Supplementary Information. [file 41598_2024_62335_MOESM1_ESM.docx]
